# Splash erosion and surface deformation following a drop impact on the soil with different hydrophobicity levels and moisture content

**DOI:** 10.1371/journal.pone.0285611

**Published:** 2023-05-12

**Authors:** Agata Sochan, Michał Beczek, Rafał Mazur, Cezary Polakowski, Magdalena Ryżak, Andrzej Bieganowski

**Affiliations:** Institute of Agrophysics, Polish Academy of Sciences, Lublin, Poland; Jinan University, CHINA

## Abstract

The splash phenomenon and the scale of the surface deformation of post-fire soils in the variants of various hydrophobicity and moisture content were studied. Splash erosion is the result of the impact of a single water drop and was analysed using high-speed cameras, while the surface deformation was parameterized using a structured light scanner. The extremely water-repellent variant (dry_V) showed distinct differences, expressed primarily in the number of ejected particles, which was 2.5 times higher than in the four soils with lower levels of hydrophobicity. It was also observed that as a result of the drop impact onto an extremely hydrophobic soil surface, a form known as liquid marble was created inside the crater. Soil moisture content determined the manner, scale and dynamics of the splash erosion. In the case of wet soils, the phenomenon proceeded up to five times faster, and as a result of the drop impact, a large number of fine particles were ejected, which reached nearly twice the velocities and three times the displacement distances compared to the dry soil group. However, the particles and/or aggregate splashed on the dry samples were larger, which also translated into the formation of craters up to twice as extensive as those in the wet soils.

## 1. Introduction

The water repellency (hydrophobicity) of soils is one of the most important soil characteristics, as it determines the physical, chemical and biological properties, as well as the agricultural use of soils [1]. Preferential paths may occur in water-repellent soils, which exert an adverse effect on the soil buffer’s ability to retain fertilizers and pesticides, and consequently, may lead to increased contamination of groundwater [2]. Additionally, this type of water flow results in the uneven distribution of moisture in the soil profile [3]. Irregular flow and moisture patterns have been observed in water-repellent, sandy soils [4], loam soils [5], clay soils [6] and also organic soils [7]. Furthermore, as emphasized by many authors, seed germination and plant growth are impaired in hydrophobic soils, which in turn, can lead to lower yields [8].

Soil water repellency (SWR) is a property that can affect soil susceptibility to erosion, e.g., by changing aggregate stability, reducing infiltration capacity or increasing overland flow, and can occur in soils with wide ranging land use types and climates [9–12]. Soil hydrophobicity impacts, among other things, water erosion, as it provides readily available loose material that can be transported on the water surface [13–15].

Fires can influence the degree of soil hydrophobicity [11, 16, 17]. In this case, an increase or decrease in SWR can depend on initial soil hydrophobicity and fire severity [18]. In addition, the distribution of hydrophobicity can vary in the soil profile. The greatest fire effects occur in the top soil layer, whereas at depth more than 15 cm, soil heating is minimal, mainly due to the poor thermal conductivity of the soil, especially in dry soils, and the lack of O_2_ for combustion [16]. It has now been proven that fire-inducted soil hydrophobicity may promote rainfall detachment and soil loss through water erosion [2, 9, 11, 19]. Leighton-Boyce et al. [20] demonstrated that on water-repellent soils, which are typical in post-fire landscapes, slope wash was up to 23 times higher than wettable soil. Moreover, Müller et al. [8] found that large soil losses can occur on hydrophobic agricultural soil and, therefore, SWR should be considered in hydrological modelling, as it has a significant impact on water movement and erosion. However, ash formed from the combustion of vegetation and litter layers reduces runoff and protects the soil from splash and sheet erosion, especially in the period immediately after fires [14, 21]. Novara et al. [22] concluded that the redistribution of soil organic carbon by water erosion across slopes is accelerated after forest fires, and contributes to the degradation of slope soils, causing organic matter enrichment of valley bottom soils. In addition, the preferential loss of silt and clay on slopes led to higher losses of organic carbon, total nitrogen and other macronutrients from water-repellent soils [15].

Soil hydrophobicity occurs naturally in many forests, especially in sandy acidic soils, but also in alkaline soils with pine vegetation [10, 23, 24]. The high temperatures during a fire cause organic substances to vaporize and migrate downwards into the soil, where they condense and coat mineral particles at cooler temperatures [25, 26]. Doerr et al. [2] presented specific fire temperature limits which cause a change in soil surface properties: at 175–200℃, soil hydrophobicity increased; at around 250℃ hydrophobic substances became permanently attached to the soil particles, while, in the case of higher temperatures, above 270–300℃, hydrophobicity decreased.

Despite the many studies of soil hydrophobicity, there are still major research gaps. This is especially true for studies at the meso- and micro-scales, which are important in terms of gaining a better understanding of the surface erosion processes and predicting the spatial variation of this phenomenon and its impact on macro-scales [12].

Therefore, the present study investigated the phenomenon of splash erosion caused by a single drop impact (a micro-scale measurement) occurring on soils with different levels of hydrophobicity and modified by high temperatures (post-fire soils). The aim of the study was to present the course and basic mechanisms of the splash phenomenon, as well as its effects (i.e., to determine the extent of surface deformation) on soils with different hydrophobicity, combined with different moisture content.

## 2. Material and methods

Soil samples were collected from the A-horizon of *Mollic Gleysol*, from a depth of 5–15 cm and from the location 50˚22’N, 23˚39’E, in the Eastern Roztocze, Poland. The authors declare that no specific permissions were required for these sampling location and confirm that the field studies did not involve endangered or protected species. This soil was selected on the basis of previous experiments, and the criterion for selection was the possibility of obtaining a wide range of hydrophobicity after fire simulation with different intensities. The main physicochemical properties of the soil were investigated by conventional methods and are presented by Boguta and Sokołowska [27]. The soil was air-dried and sieved through a 2 mm mesh sieve.

The change in soil surface hydrophobicity was obtained by fire simulation, i.e., keeping the soil sample at a high temperature for a certain period of time in a furnace. The degree of soil hydrophobicity was determined using the water drop penetration time (WDPT) test, conducted by placing five drops of distilled water onto the surface of a soil sample and recording the time taken for them to complete infiltration [28]. SWR classes were distinguished following Bisdom et al. [28]: Class I (wettable): infiltration within 5 s; Class II (slightly water-repellent): 5 s < WDPT ≤ 60 s; Class III (strongly water-repellent): 60 s < WDPT ≤ 600 s; Class IV (severely water-repellent): 600 s < WDPT ≤ 3600 s; Class V (extremely water-repellent): WDPT > 1 h. In this last case (Class V), time measurement was discontinued after 3 h, hence the small value of the standard deviation shown in Table 1. The control was a sample of an air-dry soil, not subjected to a high temperature.

**Table 1:** WDPT (with standard deviations), SWR and pH (H_2_O) for five selected soil hydrophobicity levels.

Hydrophobicity level	High temperature conditions (min; ˚C)	WDPT (s)	SWR	pH
dry_I	control	2.78 ±0.38	wettable	7.89
dry_II	1; 150	47.75 ±1.98	slightly water repellent	7.87
dry_III	5; 200	445.71 ±9.91	strong water repellent	7.66
dry_IV	10; 175	2925.88 ±21.57	severely water repellent	7.65
dry_V	10; 250	10800.00 ±0.01	extremely water repellent	7.03

The experimentally selected times and temperatures of the fire simulation made it possible to differentiate the soil samples into different classes of water repellency, depending on the WDPT result. A summary of the five selected levels is presented in Table 1. In addition, pH was measured electrochemically in H_2_O, using a digital pH meter (Radiometer Copenhagen) in each class of hydrophobicity.

The study was conducted for two variants, i) dry samples (divided into five water-repellency classes) and ii) wet samples (characterized by different levels of hydrophobicity in the dry variants). The soil material was placed in aluminium rings with a diameter of 40 mm and height of 10 mm, protected from underneath with chiffon. In the case of wet samples, the soil material was left to moisten by capillary rise. The samples were placed in a vessel where the water level was at the height of the ring. The procedure was continued for at least 24 h until the topsoil layer was wetted. These samples were termed wet, with a number of them corresponding to the water repellency class of dry soil (samples from wet_I to wet_IV), despite the fact that after wetting, they no longer showed a variation in the hydrophobicity level. Since the soil dry_V was extremely water repellent (Table 1), wetting this sample was impossible. Therefore, there are no data for this wet soil variant. Measurements for each sub-sample were carried out in 10 replicates.

Splash phenomenon was recorded using a set of three synchronized, high-speed cameras, Phantom Miro M310 (Vision Research, USA) with a speed of 3256 fps at the highest available resolution (1280 x 800 px). Two cameras were positioned 0.8 m from the sample; the angle between them was 60º and the optical axis was set at the same height as the soil surface. To determine the position of the cameras in relation to one other and the soil sample, the system was calibrated according to Beczek et al. [29]. The third camera was placed 0.6 m above the sample and checked the centricity of the impacts, as well as the dynamics of the crater formation. The recordings and analyses were performed using Phantom Camera Control (Vision Research, USA) software.

Water drops were created using the peristaltic pump, Aqua-trend TH15 (Aqua-trend, Poland). The glass capillary with an internal diameter of 1.5 mm was used for the formation of the drops with a diameter of 4.2 mm (SD = 0.02). Single drops fell freely on the soil sample from a height of 1.5 m. The final velocity of each drop was read from the recordings (the distance that the drop travelled during the last 10 frames before hitting the soil surface was used for the calculations) and this velocity was 4.98 m·s^-1^ (SD = 0.01). The Weber number (*We* = *ρv*^2^ *dσ*^−1^) was 1425, where: *ρ–*water density (kg·m^-3^), *σ*–surface tension for a water–air interface (N·m^-1^), *d–*diameter of the drop (m), and *v–*velocity of the drop (m·s^-1^), respectively.

A Volumetric 3D Particle Tracking Velocimetry (Volumetric 3D PTV) module with Dantec DynamicStudio (Dantec Dynamics, Denmark) software was used to track the movement of the splashed material in subsequent video frames and to create the flight trajectories of the ejected particles. Based on this, the number of detached soil particles were determined, as well as the velocities, displacement distances, altitudes (the maximal height reached by the ejected particles) and ejection angles for each of them [28]. Due to the nature of the phenomenon, as well as the limitations of the Volumetric 3D PTV in the process of tracking particles in space, in the case of splash on extremely water repellent soil (dry_V), a certain group of ejected particles could not be analysed in detail. After the drop impact, some of the finest particles were ejected as a group of dense cloud at low height, which made it impossible to identify and track these particles in an overly large concentration; there was also mutual obscuring of the grains. All large particles and soil aggregates were correctly tracked, even against the background of this cloud, while the finest particles were analysed these were separated from the group and were displaced outside the area of greatest concentration of the particles’ cloud.

**Fig 1:**
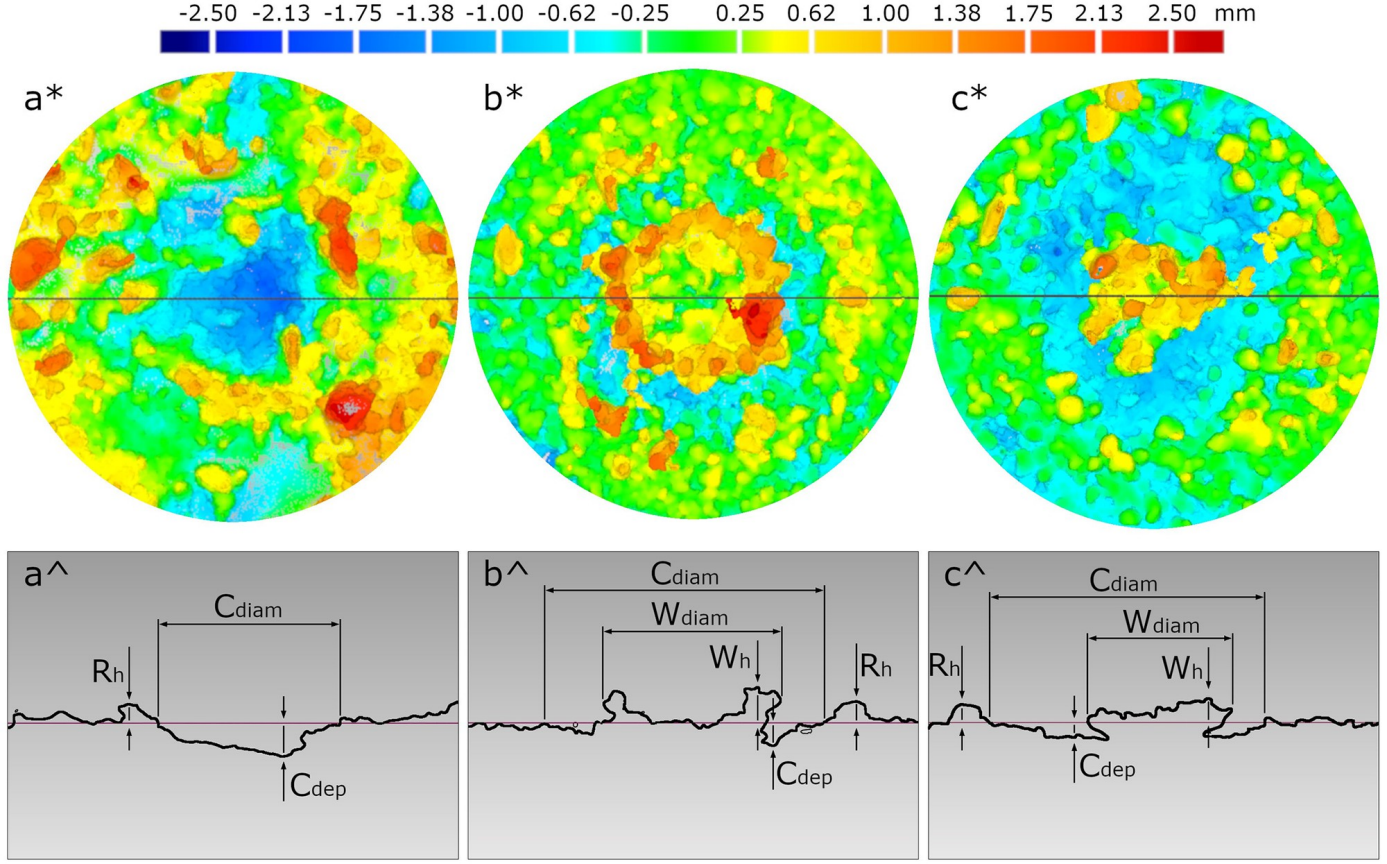
Scans of the soil surface after raindrop impact (* top images) and cross sections (^ bottom images) for three different soil samples: a) sample wet_II, b)sample dry_II, c) sample dry_V, obtained using Geomagic Control software. The horizontal lines marked on the top scans show where the cross sections were made. The cross sections show how the dimensions of the individual forms were determined: C_diam_−diameter of the crater, C_dep_−depth of the crater, R_h_−height of the rim, W_diam_−diameter of the wetted surface and W_h_−height of the wetted surface.

To determine the magnitude of the surface deformation caused by water drops, a Scan3D UNIVERSE 10 MPiX structural light scanner (Smarttech 3d, Poland) was used. The measurement of a single sample consisted of six scans carried out around the sample with rotation in 60 increments. As a result, points clouds were obtained, which were calibrated and converted into triangular meshes using Smarttech3Dmeasure software (Smarttech, Poland). Deformation dimensions were then quantified by analysing the triangular meshes in Geomagic Control software (3D Systems, USA). Based on the three-dimensional scans and the subsequent surface sections (Fig 1), the crater diameter (C_diam_) and crater depth (C_dep_), as well as the height of the rim (R_h_) were determined. In addition, the wetted surface dimensions, such as diameter (W_diam_) and height (W_h_), were determined only in the dry samples (as they were not visible in the wetted soils). The wetted surface refers to the area over which the soil particles have been wetted and bound by water from a single drop impact [30].

Statistical analyses were performed using Statistica 13.1 software. Shapiro-Wilk normality tests, one-way analysis of variance (ANOVA) and Tukey’s post-hoc test with a significance level α = 0.05 were performed.

## 3. Results

### 3.1. Splash erosion on soils with varying hydrophobicity and moisture content

**Fig 2:**
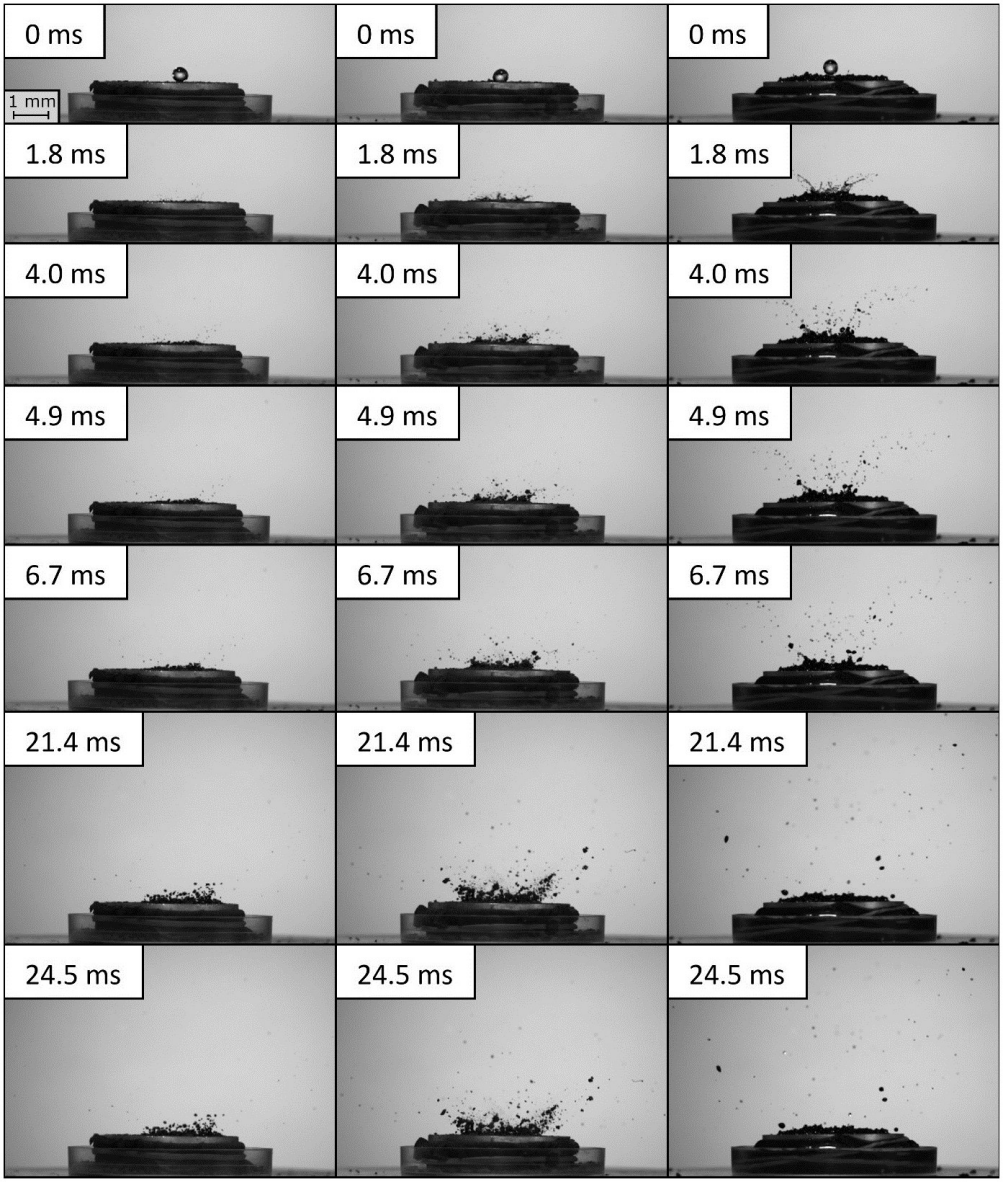
Splash phenomenon on investigated soil samples with various hydrophobicity and/or moisture content (left column–sample dry_II; middle column–sample dry_V; right column–sample wet_II), at seven different time intervals.

The splash phenomenon on selected soil samples: slightly hydrophobic dry soil (left column), extremely hydrophobic dry soil (middle column) and wet soil (right column) at seven different time intervals, counted from the moment of the drop impact is presented on Fig 2.

The splash phenomenon on soil with low degree of hydrophobicity (Fig 2 left column—sample dry_II) lasted shorter than in the case of soil with high degrees of hydrophobicity (Fig 2 middle column—sample dry_V). The first soil particles, detached from the surface of the dry_II soil, were observable at around 6 ms (in the previous intervals, mainly water droplets were splashed). However, the last ejected particles or their aggregates were observed at around 22 ms. In the case of dry_V soil, the first soil particles or their aggregates were detached from the soil surface at around 2 ms, and the last particles at 25 ms. In the case of this sample, the aforementioned cloud of fine particles can be seen in the above images (e.g., the central part of the t = 1.8 ms image).

In the case of wet samples, the splash phenomenon was very dynamic (Fig 2 right column). As a result of the drop impact, soil material was ejected intensely along with water droplets, which lasted less than 5 ms. The first soil particles or their aggregates were detached at approximately 2 ms, and the last particles/aggregates at around 7 ms. In addition, the ejected particles reached high velocities and high altitudes, as well as significant displacement distances (Fig 2 right column).

Due to the different mechanisms causing splash for wet and dry samples, the following subsections present the results for these groups separately.

#### 3.1.1. Splash phenomenon and surface deformation on dry soil

The average values of the quantities describing soil splash and surface deformation, obtained after water drop impact on the surface of dry soils with different degrees of hydrophobicity are presented in Table 2.

**Table 2:** Mean values (with standard deviations) determined for the parameters of ejected particles (grey column) and surface deformation formed after the water drop hit the surface of dry soils with five levels of hydrophobicity.

Hydropho-bicity level	Parameters of ejected particles					Parameters of surface deformation		
	number	velocity [m/s]	displa-cement distance [mm]	ejection angle [º]	altitude [mm]	crater depth [mm]	crater diameter [mm]	rim height [mm]
dry_I	82.5 ±10.3 a	1.6 ±0.2 a	67.2 ±15.6 a	40.7 ±5.2 a	11.8 ±2.9 a	0.9 ±0.2 a	13.4 ±1.4 a	0.8 ±0.3 a
dry_II	77.1 ±18.0 a	1.7 ±0.3 a	88.0 ±21.1 ab	33.0 ±3.0 b	13.1 ±2.2 ab	1.2 ±0.3 ab	16.0 ±1.6 bc	0.7 ±0.3 a
dry_III	79.0 ±11.9 a	1.7 ±0.1 a	92.2 ±16.6 b	37.0 ±3.1 ab	15.6 ±2.0 bc	1.0 ±0.3 ab	14.3 ±1.5 ac	0.7 ±0.2 a
dry_IV	83.4 ±17.0 a	1.8 ±0.4 a	94.2 ±11.0 b	36.8 ±3.1 ab	16.2 ±2.7 c	1.3 ±0.2 b	15.8 ±1.3 bc	0.6 ±0.3 a
dry_V	206.9 ±30.7 b	1.8 ±0.2 a	89.1 ±17.1 b	40.8 ±3.5 a	14.1 ±1.8 abc	1.1 ±0.2 ab	16.6 ±1.5 b	0.7 ±0.3 a

Letters indicate the statistical significance of the differences

An analysis of the results obtained for successive hydrophobicity levels in the dry samples did not show significant differences across most of the analysed parameters of the ejected particles (Table 2 - column marked in grey). In the case of altitude and ejection angle parameters, no trend was observed in the values of these quantities, obtained with increasing soil hydrophobicity. The greatest and statistically significant difference was recorded for the number of particles, whose value for the dry_V sample was even 2.5 times higher than for the other samples. This sample proved to be particularly interesting in the subsequent analysis.

**Fig 3:**
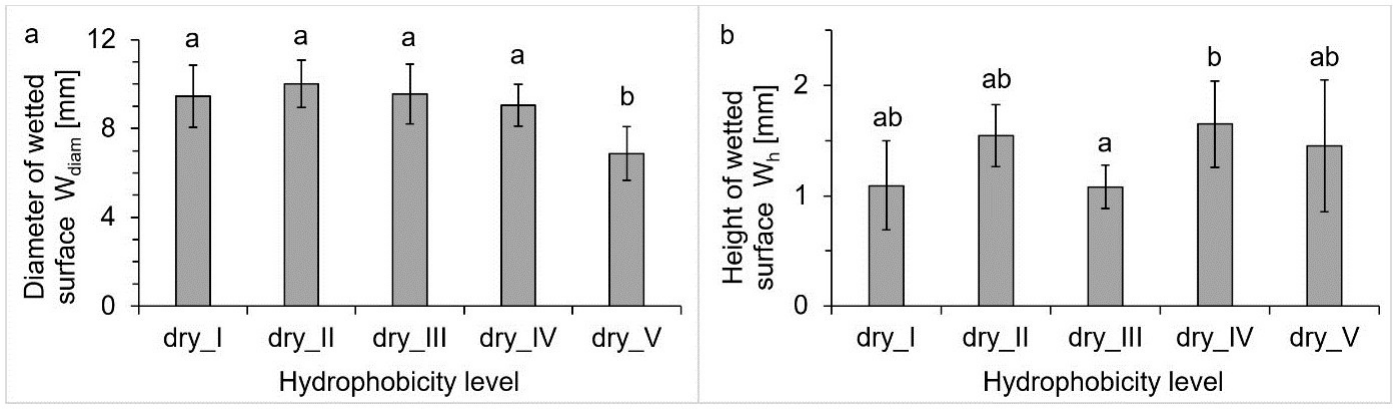
Diameter (a) and height (b) of the wetted surface formed after drop impact on the five dry soil hydrophobicity levels. Error bars on the graph represent the standard deviation; letters indicate the statistical significance of the differences.

Similarly, as regards the three parameters describing the scale of surface deformation, there was no trend in the obtained values for dry soils with successive hydrophobicity levels. However, clear differences in the additional forms created inside the crater were observed. These forms were generally called the wetted surface since they formed directly in the area of drop interaction. These different forms were previously shown in the cross sections of Fig 1b, 1c. Based on the scans, the wetted surface parameters were determined for each of the dry soil hydrophobicity levels. The results are summarized in Fig 3.

The differences in the wetted surface according to the soil hydrophobicity level, presented in Fig 1b and 1c, were confirmed, in particular, by the determination of the diameter of this form. The value of this parameter, determined for the dry_V sample, was 1.5 times smaller than for the other soils with lower levels of hydrophobicity. The height of the wetted surface often depended on the presence of a single aggregate or sand particle, hence the greater variability of the results obtained, and no trend (Fig 3b). With such small heights of this form, the size of the aggregate and/or single grains of the sand fraction resulted in a considerable difference in the reading of this dimension.

It is worth noting that the course of the phenomenon of splash and crater formation on the extremely hydrophobic sample, proceeded differently than in all the other cases. The statistically significant difference in the diameter of the wetted surface for the dry_V sample (Fig 3a) was the result of the formation of a different form than on the other dry soils. Following the drop impact, there was a process of water attracting nearby soil particles and pulling them further and further inward, thus reducing the size of the wetted surface formed. On the recorded films, the effect of "trapping" the drop inside the surrounding soil particles was observed, while the outside of the form remained dry. The average formation time of this surface was 53 ms, while the time required for water to soak into the other dry samples ranged from 21.9 to 27.4 ms, depending on the soil hydrophobicity level.

#### 3.1.2. Splash phenomenon and surface deformation on wet soil

The average values of the quantities describing soil splash and surface deformation, obtained after water drop impact on the surface of the group of wet soils are presented in Table 3.

**Table 3:** Mean values (with standard deviations) determined for the parameters of the ejected particles (grey column) and surface deformation formed after the water drop hit the surface of the wet soils.

Wet soils	Parameters of ejected particles					Parameters of surface deformation		
	number	velocity [m/s]	displa-cement distance [mm]	ejection angle [º]	altitude [mm]	crater depth [mm]	crater diameter [mm]	rim height [mm]
wet_I	177.6 ±25.5 a	2.8 ±0.2 a	200.4 ±23.2 a	32.5 ±4.3 a	30.8 ±5.1 a	1.8 ±0.4 a	7.7 ±1.3 a	1.2 ±0.5 a
wet_II	232.8 ±23.4 b	2.8 ±0.2 a	214.4 ±19.4 a	33.0 ±3.5 a	33.3 ±3.1 a	1.8 ±0.2 a	8.7 ±1.0 a	1.2 ±0.3 a
wet_III	177.6 ±18.1 a	2.7 ±0.2 a	196.6 ±33.8 a	33.3 ±4.3 a	31.8 ±6.8 a	1.8 ±0.4 a	7.2 ±1.1 a	1.0 ±0.4 a
wet_IV	173.6 ±41.2 a	2.8 ±0.4 a	181.5 ±30.8 a	32.5 ±3.8 a	29.4 ±4.7 a	1.6 ±0.5 a	8.0 ±1.8 a	1.3 ±0.5 a

Letters indicate the statistical significance of the differences

An analysis of the results obtained for the four wet samples, (which had different levels of hydrophobicity before wetting), showed no significant differences in the values of the ejected particle parameters (Table 3 - column marked in grey). Similarly, in the case of the parameters describing crater and rim size, no trend was found in the obtained values for the wet soils (from wet_I to wet_IV).

In addition, Table 4 shows the average values of the quantities describing soil splash and surface deformation, obtained for both moisture groups: i) dry and ii) wet soils.

**Table 4:** Mean values (with standard deviations) determined for the parameters of the ejected particles (grey column) and surface deformation formed after the water drop hit the surface of the two groups of dry and wet soils.

Soil moisture	Parameters of ejected particles					Parameters of surface deformation		
	number	velocity [m/s]	displa-cement distance [mm]	ejection angle [º]	altitude [mm]	crater depth [mm]	crater diameter [mm]	rim height [mm]
dry	106 ±54 a	1.7 ±0.3 a	86.1 ±18.7 a	37.7 ±4.6 a	14.1 ±2.8 a	1.1 ±0.3 a	15.2 ±1.8 a	0.7 ±0.3 a
wet	190 ±37 b	2.8 ±0.3 b	198.2 ±28.9 b	32.8 ±3.8 b	31.3 ±5.1 b	1.7 ±0.4 b	7.9 ±1.4 b	1.2 ±0.4 b

Letters indicate the statistical significance of the differences

The statistical analysis, in which soil moisture was the differentiating factor showed significant differences across all quantities of the ejected particles (Table 4). The scale index, determined as the ratio of the mean value for the wet group, divided by the mean value for the dry group, was 1.6, 2.3 and 2.2 for the parameters’ velocity, displacement distance and altitude, respectively. In the case of the number of particles, the above ratio was also high (1.8), however, it should be kept in mind that in the dry_V samples, it was not possible to count all the particles accurately, as they formed too dense a cloud.

Similarly, all values of the determined surface deformation magnitudes differed statistically significantly across the moisture variants. The average values of crater depth and rim height in both groups were low, at 1.1 and 1.8 mm (C_dep_ for the dry and wet groups) and 0.7 and 1.2 mm (R_h_ for the dry and wet groups), respectively. In contrast, the crater diameter was nearly two times larger in the case of the dry samples, as compared with the wet samples.

## 4. Discussion

### 4.1. Effect of fire conditions on soil properties

Using the specific fire temperature limits presented by Doerr et al. [2], soils with varying levels of hydrophobicity were obtained in our experiment. However, it should be noted that an increase in fire temperature did not linearly affect soil hydrophobicity. It is also noteworthy that the applied fire temperature in variant dry_IV was lower than in variant dry_III (Table 1), yet the final hydrophobicity was higher. This may have been influenced by the twice longer burning time applied. Thus, it can be concluded that the final degree of soil hydrophobicity depended jointly on the duration and temperature of the fire. A similar observation was noted by Ngole-Jeme [26], who stated that a fire of a lower temperature and longer duration had a similar effect to a fire of a higher temperature and shorter duration.

During the burning process, other soil properties also changed. It is worth noting that attempts at re-wetting the extremely hydrophobic material were unsuccessful. Soil variant dry_V, when left to soak, remained dry and non-wetting. A similar phenomenon was also observed by Doerr et al. [2], who concluded that re-wetting does not always restore the soil to its original moisture content.

There was also a noticeably greater dusting during the splash on the dry_V sample (presence of a dense cloud of finest particles) than in the control sample. Details of the effects of fire on the physical properties of soils, including moisture content, aggregate stability and texture have already been described [11, 17, 24].

In addition, fire also affected the soil chemical properties, including the pH (Table 1), which decreased with increasing fire intensity, from 7.9 to 7.0. Previously published results on the fire effects on soil pH are contradictory. An increase in pH with increasing fire temperature was reported by [31, 32], while [16, 33] reported a pH decrease. As summarized by Ngole-Jeme [26], most of the studies that reported a decrease in pH were conducted in a laboratory, which does not take into account the effect of ash from burning plant residues in the soil. This was also the case in our laboratory analyses.

### 4.2. Effect of soil hydrophobicity on splash erosion

On the hydrophobic surfaces, the drop impact could cause either a prompt splash followed by bouncing on the surface (low Weber number) or a crown splash with satellite droplets ejected from the lamella edge (high Weber number) [34]. On the hydrophilic surface, on the other hand, the drop soaks in after impact and adheres because of the capillary force at the contact line. Zhang et al. [34] also emphasized that despite the above and regardless of the surface type, the splash gradually transitions from prompt to crown splash, depending on the energy of the falling drop.

In the case of our experiments, the crown splash, induced by a drop of 0.5 mJ energy, was dominant; the determined We value of 1425 falls within the range, corresponding to the occurrence of this type of splash (200<We<2000) and presented previously by Pan and Hung [35]. Every single water drop impacting on the surface of the soil samples across all the hydrophobicity and moisture variants we analysed, caused a significant scale of splash, expressed, among others, by the number of ejected particles, which ranged from 80 to more than 200 (Table 4). As already mentioned, this number did not take into account some of the finest particles, which rose as a dense cloud from the surface of dry_V sample. The ejected, counted particles were displaced at a distance of 65 to 215 mm, depending on the variant of soil moisture content, which resulted in different mechanisms of detached soil material (Table 4). An additional effect of drop impact was the formation of a micro-crater on the soil surface. Its diameter again depended on the moisture variant and ranged from around 7 mm (for wet samples) to as much as 16.5 mm (for dry samples), exceeding the diameter of the falling drop significantly.

The scale and dynamics of the splash depended on the type of surface with which the drop interacted. The greatest differences were recorded for the extremely hydrophobic variant (dry_V). Two parameters were particular outliers: i) the number of ejected particles was around 2.5 times larger than for the other dry soils (Table 2) and ii) the diameter of the wetted surface was nearly 1.5 times smaller than the rest of the values (Figs 1b, 1c and 3a). As Heydari et al. [36] concluded, the destruction of the soil organic-mineral structure that occurs during high-intensity fires leads to increased closure of the soil pores, which consequently, reduces the soil’s ability to retain water and increases the likelihood of runoff and surface erosion. Ahn et al. [9], using model glass bead systems, showed that beads with a hydrophobic surface were splashed more frequently and over greater distances than those with a hydrophilic surface. Our experiments confirmed the aforementioned conclusion–the average displacement distance determined for the group of hydrophobic soils was 90.9 mm, while, in the case of wettable soil, this parameter was less (67.2 mm). Moreover, Fox et al. [11] inferred greater splash erosion on the burned samples than on the control sample. It is difficult to compare our results unequivocally because, in the Fox et al. experiment, the soil sample was subjected to a 30-minute rainfall simulation, then all the transferred material was weighed, hence quantifying the total erosion caused by the splash. Nevertheless, indirect confirmation is provided by observing the highest number of ejected particles from the extremely hydrophobic soil, as well as the formation of more extensive craters in the burned soils (the average crater diameter was 15.7 mm) than in the control sample (13.4 mm). The other analysed splash quantities (e.g., particle velocity, ejection angle, altitude) did not differ statistically significantly between soils with different hydrophobicity.

However, it is worth noting that even in the case of parameters for which no statistically significant differences were recorded between successive hydrophobicity variants (soils from dry_I to dry_V), clear differences were evident amongst groups of dry and wet samples (Table 4). Thus, it can be concluded that the variation in soil moisture determined the course of the entire splash phenomenon, from the type and size of the ejected particles (Fig 2), through to their number, velocity and displacement distances, and to the scale of surface deformation (Fig 1 and Table 4). More particles were ejected from the surface of wet soils than dry soils (not including part of the finest particles).

In addition, the particles from the wet soils reached higher velocities than those from the dry soils (2.8 and 1.7 m/s, respectively), and consequently, were splashed higher (31 and 14 mm, respectively) and displaced further (198 and 86 mm, respectively). However, the particles from the wet samples were significantly smaller, as illustrated in Fig 2. Thus, it can be concluded that due to the presence of water in these samples, there is an additional cohesive force at the particle-water-particle interface, which hinders the separation and ejection of the material. Beczek et al. [37] proved that as a result of splash wet soil material, the liquid phase (i.e., water coming from a drop, broken down into a large number of smaller droplets) can be ejected, but also a mixture of water and soil, which was the case in our experiment. The tiny droplets of the ejected water-soil mixture reached similar velocity and displacement distances as those of pure water droplets, breaking on a model glass bead system, as observed by Sochan et al. [38].

Considering the splash phenomenon, based on the transferred mass, it should be noted that the ejected particles (and/or aggregates) from the dry samples were significantly larger than those from the wet soils (Fig 2). The significant number of splashed particles, as well as their size, directly translated into the size of the craters formed. It was observed that in the dry samples, the material was ejected largely by the mass of the sample wetted by the drop, which, as it spilled, transferred the force used to eject the particles slightly further from the centre of the drop impact. The subsequent splash occurred as a result of the collision of the particles (ejected dry particles were observed). Moreover, the formation of less extensive micro-craters on the surface of the wet samples was associated with the high-water saturation in this group of samples. The diameter of the craters, formed on wet samples, was nearly half the size compared to the dry samples (Fig 1 and Table 4). When measuring the depth of craters, the presence of water affected the final result to a lesser extent (Table 4).

Despite the significant differences in the results obtained for the two soil moisture variants, it is difficult to compare them directly with one other, due to the different mechanisms of the phenomenon. In addition, given the different parameters measured, it is difficult to indicate which splash was greater. In the context of the mass transferred and the scale of surface deformation, a larger splash was formed on dry soils. Due to the number of ejected particles, the magnitude of the splash was greater on the wet samples. However, the uncountable part of the finest particles ejected from the surface of the dry samples should also be noted. Similar problems regarding the tracking of the ejected particles in the dense cloud group were observed by Tsujido et al. [39], who investigated the high-velocity impact onto a quartz sand bed. In that case, the ejecta curtain did not allow to observe the single displaced grains, and an analysis was only performed on the separated particles at the outer edge of the curtain. Deboeuf et al. [40] observed the impact of steel spheres impacting on the granular bed (glass beads with an average diameter of around 0.4 mm) using high-speed cameras. Similarly, as above, the dense corona of the grains was difficult to analyse in the captured 2D images of the single grains. Deboeuf et al. [40] proposed an estimation of the number of ejected grains on the basis of the shape/dimensions of the corona and the assumed layer thickness of this, but highlighted the issue of the mutual obstruction of the grains in the corona. As stated, the grains ejecting upwards as a dense cloud at low heights, were characterized by negligible ejection velocity, thus absorbing an insignificant element of the kinetic energy of the impacting drop, i.e., around 0.3% [40]. Based on the above examples, it can be assumed that in our case, the group of finest particles, ejected as a cloud, may be marginal in relation to the splash phenomenon. Although such particles may be ejected in large numbers, due to their small size, their total mass is likely to be negligible compared to the displaced large grains and soil aggregates.

### 4.3. Occurrence of liquid marble

The formation of a wetted surface was observed on the dry soils as a result of drop impact. Its form differed significantly, depending on the degree of soil hydrophobicity. The largest and statistically significant differences were recorded for the extremely water repellent soil (Fig 3a). Moreover, while in the other dry soils, the footprint of the drop impact was termed a wetted surface, in the case of the dry_V sample, the soil material did not wet at all, but only "coated" the drop standing on the surface. This form has already been described in the literature as liquid marble [41–43]. It is a fairly stable structure, consisting of a shell of hydrophobic particles surrounding a liquid core, i.e., a water drop. The hydrophobic coating insulates the liquid from the substrate in which liquid marble exhibits minimal adhesion to the surface [41, 44].

In a model study, Atherton et al. [45] showed that depending on the arrangement of the layers of hydrophobic and hydrophilic beads, the liquid marble may not only form on a completely hydrophobic substrate, but hydrophobic beads could also form a coating around the outside of the drop, while hydrophilic beads may be absorbed into the drop. Atherton et al. concluded that wherever there was a loose hydrophobic layer, the drop formed a liquid marble, while only in the case of a completely hydrophilic substrate did the drop have the opportunity to soak into the bead pack.

An analysis of the morphology of liquid marble, formed from hydrophobic silica powder, was presented by Nguyen et al. [46]. Using a confocal microscope, Nguyen et al. imaged a coating that consisted of multiple layers of fine material. The particles, which were several nanometres in size, reduced the contact between the drop and the surface, and thus increased the elasticity of the form. Nguyen et al. concluded that for highly hydrophobic particles, the dominant forces maintaining the liquid marble structure were short-range, attractive, van der Waals forces between the hydrophobic particles. Hiemenz and Rajagopalan [47] maintained that due to the lack of hydrogen bonding between the hydrophobic particles and the water, as well as the strong hydrogen bonding between the water molecules, water withdraws from the inter-particle region, resulting in a larger particle-particle contact area. Accordingly, Nguyen et al. [46] speculated that the forces of attraction between hydrophobic particles play a dominant role in maintaining the stability of liquid marble, in contrast to the interactions between particles and the liquid core, which play a minor role in maintaining the integrity of the form.

Although, liquid marbles can have a wide range of application, from industry to medicine [42], in the context of hydrological soil behaviour, their occurrence can have adverse effects. According to Hamlett et al. [19], the hydrophobic top layer increased splash erosion, due to liquid marble formation, but this effect did not penetrate the lower layers of the soil. Nevertheless, the presence of hydrophobic particles just below the soil surface can result in the simultaneous erosion of multiple matrix layers.

## 5. Conclusions

On the basis of the experimental results obtained, particularly for wettable (dry_I), slightly water repellent (dry_II), strongly water repellent (dry_III) and severely water repellent (dry_IV) soils, it was found that the degree of soil hydrophobicity was not the main determinant of the splash phenomenon, formed under the impact of a single raindrop. Only the extremely water repellent soil (dry_V) showed distinct and statistically significant differences, expressed primarily in the number of ejected particles, which was 2.5 times higher than in soils with lower hydrophobicity level. It was also observed that as a result of the drop impact onto an extremely hydrophobic soil surface, a different form of surface deformation was produced than on other dry samples. Thus, a micro-crater was formed on soils with a lower degree of hydrophobicity, in the centre of which a wetted surface was observed, while in the dry_V sample, liquid marble was formed inside the crater.

Soil moisture content significantly determined the manner, scale and dynamics of splash erosion. In the case of the group of four wet soils, formed by wetting samples with different initial degrees of hydrophobicity (wet_I to wet_IV; extremely hydrophobic soil remained non-wetting), the phenomenon proceeded up to five times faster and, as a result of the drop impact, a large number of fine particles (in the form of a water-soil mixture) were ejected, which reached nearly twice the ejection velocities and three times the displacement distances, compared to the dry soil group. However, the particles and/or aggregate splashed on the dry samples were larger, and some of them were ejected by inelastic particle-particle collisions in view of which they remained completely dry. The surface deformation on dry soils was also greater, as expressed by the formation of wider craters (their diameter was two times larger than those in the wet soil group).
